# The Network Theory of Psychiatric Disorders: A Critical Assessment of the Inclusion of Environmental Factors

**DOI:** 10.3389/fpsyg.2021.623970

**Published:** 2021-02-04

**Authors:** Nina S. de Boer, Leon C. de Bruin, Jeroen J. G. Geurts, Gerrit Glas

**Affiliations:** ^1^Department of Philosophy, Radboud University, Nijmegen, Netherlands; ^2^Department of Philosophy, Vrije Universiteit Amsterdam, Amsterdam, Netherlands; ^3^Department of Anatomy and Neurosciences, Amsterdam University Medical Centers (Location VUmc), Amsterdam, Netherlands

**Keywords:** network theory, network analysis, causality, interventionism, mechanistic explanation, topological explanation, multilayer network, psychiatry

## Abstract

Borsboom and colleagues have recently proposed a “network theory” of psychiatric disorders that conceptualizes psychiatric disorders as relatively stable networks of causally interacting symptoms. They have also claimed that the network theory should include non-symptom variables such as environmental factors. How are environmental factors incorporated in the network theory, and what kind of explanations of psychiatric disorders can such an “extended” network theory provide? The aim of this article is to critically examine what explanatory strategies the network theory that includes both symptoms and environmental factors can accommodate. We first analyze how proponents of the network theory conceptualize the relations between symptoms and between symptoms and environmental factors. Their claims suggest that the network theory could provide insight into the causal mechanisms underlying psychiatric disorders. We assess these claims in light of network analysis, Woodward’s interventionist theory, and mechanistic explanation, and show that they can only be satisfied with additional assumptions and requirements. Then, we examine their claim that network characteristics may explain the dynamics of psychiatric disorders by means of a topological explanatory strategy. We argue that the network theory could accommodate topological explanations of symptom networks, but we also point out that this poses some difficulties. Finally, we suggest that a multilayer network account of psychiatric disorders might allow for the integration of symptoms and non-symptom factors related to psychiatric disorders and could accommodate both causal/mechanistic and topological explanations.

## Introduction

How should we explain why and how symptoms of psychiatric disorders arise? According to a long-established view, this can be done by conceptualizing symptoms as the effects of a common cause. Proponents of this view (henceforth referred to as the *traditional view*) often assume that the common cause in question is neurobiological in nature, and thus (often implicitly) endorse the idea that psychiatric disorders can be explained in terms of lower-level, (neuro)biological properties. The influence of this view in the scientific debate is most convincingly exemplified by an article published by the former heads of the National Institute of Mental Health (NIMH) in *Science* titled “Brain disorders?, Precisely”, stating that new diagnostics will likely redefine mental disorders as “brain circuit disorders” (Insel and Cuthbert, 2015). Their claims are in line with the NIMH’s Research Domain Criteria (RDoC) initiative, a now widely adopted framework that aims to transform our current diagnostic frameworks for psychiatric disorder classification into a biological system that “conceptualizes mental illnesses as brain disorders” (Insel et al., 2010, p. 749). Despite the influence of the traditional view, however, there is not much empirical evidence to support it. As Adam (2013 p. 417) puts it: “Despite decades of work, the genetic, metabolic, and cellular signatures of almost all mental syndromes remain largely a mystery.” To illustrate, a recent meta-analysis on 73 potential biomarkers for obsessive-compulsive disorder demonstrated that none had sufficient sensitivity or specificity (Fullana et al., 2020).

A promising alternative account of psychiatric disorders that has gained traction over the past years is the *network theory*, which conceptualizes psychiatric disorders as relatively stable networks of interacting symptoms (Borsboom, 2017; Borsboom et al., 2019a).^1^ Although network science has been around since the late twentieth century (Barabási, 2012), its application to psychopathology is fairly recent and provides a new way of understanding and explaining psychiatric disorders. Whereas proponents of the traditional view typically argue that the causes of psychiatric disorders are localizable in the brain, the network theory moves our focus from the brain to psychiatric symptoms and their relations. Proponents of the network theory (e.g., Borsboom, 2017; Borsboom et al., 2019a) have argued that the theory should not only focus on the symptom network, however, but should also include non-symptom factors relevant in the context of psychiatry, such as *environmental factors*. Examples are adverse life events, social relations, but also more pragmatic items such as external objects (e.g., gambling machines in gambling addiction, Borsboom et al., 2019a).^2^ The underlying motivation is that different factors are involved in the development and sustenance of psychiatric disorders, and that we can only properly understand and explain these disorders if we take these factors and their relation to each other into account (Kendler, 2008; Nolen-Hoeksema and Watkins, 2011). How are environmental factors incorporated in the network theory, and what kind of explanations of psychiatric disorders can such an *extended* network theory provide? Addressing these questions is important because proponents of the network theory do not just want to use network models as instruments to investigate psychiatric disorders: they want to provide a theory of what psychiatric disorders *are* (Borsboom et al., 2019b). Although they have made various claims on the role of environmental factors in the network theory and the theory’s explanatory potential, these claims would benefit from further justification.

The aim of this article is to critically examine what explanatory strategies the network theory that includes both symptoms and environmental factors can accommodate. First, we will analyze how proponents of the network theory conceptualize the relations between symptoms and between symptoms and environmental factors. We will focus primarily on the accounts of Borsboom (2017) and Borsboom et al. (2019a), since these are seminal papers on the network theory of psychiatric disorders and also make various claims on the causal and/or constitutive role of symptoms and environmental factors in relation to psychiatric disorders. Afterwards, we will examine if we can corroborate these claims using network analysis, Woodward’s interventionist theory of causation, or mechanistic explanation. Next, we will examine the claim that the network theory can explain the dynamics of psychiatric disorders by referring to the network’s characteristics by means of a topological explanatory strategy. Finally, we will introduce the *multilayer* network account of psychiatric disorders as a framework that allows for the integration of symptoms and non-symptom factors related to psychiatric disorders, and could potentially accommodate both causal/mechanistic and topological explanations.

## The Network Theory of Psychiatric Disorders

### The Symptom Network

Borsboom and colleagues make two main claims about relations in the symptom network. The first claim concerns the relations between symptoms. The network theory states that psychiatric symptoms *causally* interact with each other (Borsboom, 2017). This causal interpretation of the covariance between symptoms is justified by referring to folk psychology: they claim that it *makes sense* for certain symptoms to be causally related (Borsboom et al., 2019a).^3^ It seems to make sense, for example, that insomnia can lead to fatigue and that hallucinations can lead to the development of delusions (Kendler et al., 2011). However, those critical of the network theory could argue that intuition and sense-making are not necessarily reliable criteria for determining causality.

The second claim concerns the relation between symptoms and the psychiatric disorder in question. The network theory claims that the (causal) interactions between the symptoms themselves is *constitutive* of the disorder, rather than symptoms being caused by an underlying disorder.^4^ To illustrate the difference between these views, consider the diagnostic criteria for major depressive disorder (MDD). According to the fifth edition of the *Diagnostic and Statistical Manual of Mental Disorders*, receiving a MDD diagnosis requires at least five of the following nine symptoms to be present almost every day during the same 2 week period: (1) depressed mood, (2) diminished interest or pleasure, (3) significant weight loss or gain, (4) insomnia or hypersomnia, (5) psychomotor agitation or retardation, (6) fatigue or loss of energy, (7) feelings of worthlessness or excessive/inappropriate guilt, (8) diminished ability to think/concentrate or indecisiveness, and (9) recurrent thoughts of death/suicidal ideation (American Psychological Association, 2013).^5^ The traditional view would argue that MDD is the latent or unobserved cause of all these symptoms: to treat MDD, the disorder itself should be treated, after which the symptoms should also disappear. The network theory, however, would claim that MDD is constituted by the relatively stable configuration of causal interactions between the symptoms: to treat MDD, the symptoms should be treated directly. As argued by Borsboom (2017, p. 10): “If diagnosis involves identifying a symptom network, then treatment must involve changing or manipulating that network.” But claiming that symptoms constitute a disorder also poses some issues. Since there is considerable variation in the type of symptom combinations one can have in order to receive an MDD diagnosis, how can we claim that these diverse combinations all constitute the same disorder?^6^ This example illustrates that the network theory may benefit from justification criteria for their claims concerning causality and constitution in symptom networks.

### The Role of Environmental Factors

Proponents of the network theory have also made various claims on the role of environmental factors in psychiatric disorders. First, it is sometimes claimed that symptoms and environmental factors are causally related, but that this causal relation is different from the causal relations between symptoms. Whereas it is considered that there may be feedback loops between individual symptoms, causal connections between symptoms and environmental factors are typically presented as *unidirectional*: environmental factors affect symptoms. Indeed, environmental factors are typically presented as catalysts or *background elements* of the symptom network: symptoms can be “activated by factors external to the person” (Borsboom et al., 2019a, p. 4), but the symptom network eventually becomes self-sustaining after activation. For example, losing one’s partner may lead to a depressed mood, which can lead to insomnia, anxiety, etc. (Borsboom, 2017). It has also been claimed that environmental factors can influence and determine the strength of the relations between the symptoms (Borsboom et al., 2019a), hence directly influencing symptom-symptom relations.

The relation between environmental factors and symptoms is not only presented as causal, however. It is also claimed that environmental factors can be *constitutively* related to symptoms, to symptom-symptom relations, and to the disorder itself. This constitutive relation is presented by the claim that environmental factors can be part of the *mechanisms* that constitute the disorder: “(network structures) rest on or invoke mechanisms in the environment (Borsboom et al., 2019a, p. 8).” Concerning the constitutive role of environmental factors in symptom-symptom relations, proponents of the network theory claim that “we should expect to find interactions between symptoms to be grounded in an even more complex set of biological, social, and cultural factors involved in psychopathology” (Borsboom et al., 2019a, p. 10). To illustrate this, Borsboom and colleagues examine the role of a Roulette table in gambling addiction. They state that the relationship between excessive gambling and debt – both symptoms of gambling addiction – is realized by the gambling setups that require a monetary investment, for example, in the form of a Roulette table. If we imagine a world without Roulette tables, or with Roulette tables that are operationalized in a different way, there would not be a link between excessive gambling and debt. Hence, they claim that environmental factors (such as Roulette tables) are an integral part of the symptom-symptom relation. The network theory also argues that environmental factors can co-constitute a psychiatric disorder: “in network models (…) the environment itself may become part of the network structure, and hence part of the disorder. More or less by definition, this means that (…) cultural and historical factors as well as external mechanisms, to some extent, shape mental disorders” (Borsboom et al., 2019a, p. 8). Hence, whereas they argue that environmental factors can causally influence the symptom network, they also claim that environmental factors can be part of the disorder itself.

This demonstrates that proponents of network theory suggest various ways to interpret the relation between environmental factors and symptoms: environmental factors may cause or constitute symptoms and/or symptom-symptom connections, and may co-constitute the psychiatric disorder in question. Hence, these claims suggest that the network theory could explain the causal mechanisms underlying psychiatric disorders. Are these claims justified? How can we evaluate them? In the next section, we will assess these questions in relation to network analysis, Woodward’s interventionist theory of causation and mechanistic explanation.

## The Causal/Mechanistic Explanatory Strategy

### Network Analysis

It seems like a logical starting point to attempt to corroborate the aforementioned causal claims using statistical evidence since network theory has its origins in network analysis (Borsboom, 2008). Network analysis refers to statistical techniques that estimate (i.e., approximate “true, real-world”) networks based on patterns of covariance in empirical data. These techniques generally estimate the relations between variables as *partial* correlations, i.e., associations between two traits conditioned on the other traits in the model.^7^ A partial correlation between variables *A* and *B* in a network can be interpreted as the value of variable *A* predicting the value of variable *B*. For example, Beard et al. (2016) demonstrated a statistically significant relationship between depressed mood and diminished interest in a symptom network for individuals with a MDD diagnosis. This may indicate that mood changes in MDD predict changes in interest, and vice versa. Partial correlations between symptoms and environmental factors have been estimated in a similar fashion: studies have examined cannabis use, developmental trauma and urban environment (Isvoranu et al., 2016), sexual risk (Choi et al., 2017), and spousal loss (Fried et al., 2015) in relation to symptoms of a variety of psychiatric disorders. Some studies demonstrated that environmental factors may indeed predict symptoms (e.g., spousal loss is strongly associated with loneliness, Fried et al., 2015).

There are various reasons why we should not conflate the network theory with network analysis, however, as highlighted by Fried (2020) and Robinaugh et al. (2020). First, statistically estimating relations in a network is not a theory-neutral process: there are various choices that have to be made before one can claim that a relation is present or absent. For example, we can vary the threshold used for determining statistical significance and have to decide which regularization techniques to use to correct for false positives (Epskamp et al., 2017). Second, most statistical analyses – including all the aforementioned studies – use cross-sectional, between-subject data. Identifying a relation in a between-subject design does not necessarily provide information on whether this relation is present *within* a person (Fisher et al., 2018). Although within-subject network studies are being conducted (Bringmann et al., 2013), they still constitute the minority of the studies available. Third, the boundary between statistical network models and latent variable models is more nuanced than commonly assumed (Bringmann and Eronen, 2018). These models may be *statistically equivalent*: they may fit the same dataset equally well, meaning that they cannot provide enough evidence to promote one model over the other.

A final, important reason is that the network theory wants to do more than merely predict psychiatric disorders: it wants to provide *causal* explanations. If we know the causal processes underlying psychiatric disorders, we can come up with interventions and design suitable treatments or prevention programs accordingly. We cannot simply assume that (partial) correlations imply causal relations: covariance does not necessarily imply that one of the variables *influences* the other. As the classic example of the barometer and the storm goes: one can predict a storm using a barometer, but changing the pressure readings will not prevent the storm from happening. Relatedly, the presence of (partial) correlations does not rule out the traditional view that symptoms of psychiatric disorders have a common (brain-based) cause. Indeed, symptom covariance can still be explained under the traditional view that symptoms are caused by an underlying (neurobiological) cause. Now one could argue that causal inference techniques can be used to directly estimate *directed acyclic graphs* (DAGs), i.e., causal networks without bidirectional effects or feedback loops, using correlational data (Pearl, 2000). Indeed, DAGs have been used to study the causal relations between symptoms (Borsboom and Cramer, 2013), and between environmental factors and symptoms (Moffa et al., 2017). It is important to note, however, that these causal inference methods require certain assumptions to be satisfied. They assume that the network encodes all the causal relations between factors, that there is no unobserved confounding, and that there are no causal feedback loops.^8^ These assumptions may not be met in the context of psychiatric disorders and will be discussed in more detail in the upcoming section.

Hence, we cannot corroborate the causal claims of the network theory based on network analysis alone: although statistical models can generate findings that need to be explained, they do not have the explanatory power that the theory claims to have.

### Woodward’s Interventionist Theory of Causation

Another potential means to justify the causal claims made by proponents of the network theory is to make reference to (hypothetical) interventions. This is also alluded to by proponents of the network theory: Borsboom (2017, p. 6) argues that “such causal interaction between symptoms can be interpreted using interventionist theories of causation.” The interventionist theory of Woodward (2003) has become one of the most influential approaches to causation in the past decades. It claims that causal relations should be understood in terms of the changes that result from possible interventions: if there is a possible intervention on *X* that leads to a change in *Y*, while holding fixed all other variables that could change *Y*, then *X* causes *Y*. A good intervention meets the following criteria:

1. It causes *X*;
2. It acts as a switch for other variables that cause *X*;
3. It does not cause *Y via* any other path than *via X*; and
4. It is independent of any variable *Z* which causes *Y* and is on a directed path that does not go through *X* (Woodward, 2003, p. 98).

In this way, interventionism could be used to establish causal relationships between variables without referring to an underlying (neurobiological) common cause: if we demonstrate that an intervention on *X* changes *Y* and does not affect other variables that may cause *X* or *Y*, there is a direct effect of *X* on *Y*. Interventionism thereby allows us to make claims on the relations between variables that go beyond mere correlation. It may not always be empirically possible to construe interventions on symptoms or environmental factors, but this is not necessarily problematic: interventionism requires *hypothetical* interventions that meet the conditions mentioned above (Woodward, 2014, p. 216). So, if (hypothetical) interventions on symptoms or environmental factors can be construed which adhere to Woodward’s criteria, we can make causal claims. But are we actually able to come up with (hypothetical) interventions on psychiatric symptoms or environmental factors that adhere to these criteria? In other words, can the network theory meet all assumptions necessary to draw causal conclusions?

If we focus on symptom networks, we see that this may not be as easy as posed. First, it is uncertain whether we can truly eliminate the possibility of a common cause in symptom networks, for this requires us to know (and include) all factors that are casually related to the disorder. If not, it is possible that the causal relation is ultimately due to confounding. If we knew all relevant causal variables, we would still be left with a second problem: it is uncertain whether we can come up with *surgical* hypothetical interventions on symptoms, i.e., interventions that do not influence other variables in the network. Are we able to intervene on a symptom, while keeping other variables in the network stable? It is likely that many symptom interventions have effects on *Y* which do not go through *X* (violation of criterion 3) or influence a variable *Z*, which causes *Y* and is not on a directed path through *X* (violation of criterion 4; Romero, 2015). For example, a peer support group may not be a good surgical intervention to assess whether using medication causes a stable mood, because the peer support group may enhance one’s motivation to use medication, but may also facilitate participation in meaningful activities and interaction with helpful group members, which could influence one’s mood.^9^ One could solve this problem by allowing for *fat-handed* rather than surgical interventions, i.e., interventions that not only affect *X* and other variables on the route from *X* to *Y* but also affect variables affecting *Y* which are not on this route (Woodward, 2008, p. 209; Eberhardt, 2014; Romero, 2015). But even if we allow for this, a third question arises: can we actually take for granted that psychiatric symptoms are distinct and non-overlapping entities? It is necessary to properly define target variables in order to perform suitable interventions. Although proponents of the network theory assume that symptoms are defined at the right level of detail and specificity^10^ and “successfully identify the important components in the psychopathology network” (Borsboom, 2017, p. 7), it has also been argued that it is difficult to actually pinpoint individual mental states as suitable targets for intervention (Woodward, 2014). For example, there may be conceptual overlap between the MDD symptoms “depressed mood and diminished pleasure.” This is problematic for the application of interventionism to symptom networks: if we are unable to clearly differentiate between two symptoms, we cannot come up with an intervention that does not directly affect both.^11^ Lastly, although interventionism could account for networks that are *acyclic*, it is likely that in real life, symptoms influence each other via *feedback loops*. For example, a feedback loop may be present between insomnia, fatigue, concentration problems, and stress (insomnia causes fatigue, which causes concentration problems, which causes stress, which causes insomnia, etc.). If this would be the case, an intervention on the relation between insomnia and fatigue does not act as a switch for concentration problems and stress, thereby violating criterion 2. It may sometimes be possible to circumvent this problem by taking the temporal relations between factors into account (Dijkstra and de Bruin, 2016), but these relations are not always easy to discern. Relatedly, it is possible that symptoms are just too dependent on each other to discern their individual contributions, which hampers our ability to make claims on their individual causal contributions (this will be discussed in more detail in the next section). Hence, although proponents of the network theory argue for an interventionist interpretation of causality, the interventionist criteria which should be satisfied to call a relationship between symptoms causal cannot always be met and/or tested.

What happens when we evaluate the proposed causal relations between environmental factors and symptoms in the network theory in light of the interventionist criteria? First, as discussed previously, proponents of the network theory claim that environmental factors could unidirectionally cause symptoms and thereby serve as catalysts or background elements of the symptom network. It may be possible that such a unidirectional effect can be established more easily for some environmental factors than for individual symptoms. Indeed, for some environmental factors, it may be possible to establish the temporal order of events. For example, when we want to include adverse life events in a psychiatric disorder network, we know in some instances that they happened *before* the present-day symptoms arose. This example may run into similar problems of meeting the criteria for good interventions, however. Can we ascertain that we know all relevant causal factors, and can we ensure that (hypothetically) intervening on an environmental factor affects one symptom only? Again, removing people from a stressful home environment may, for example, affect their mood and their agitation. We could circumvent this problem if we allow for fat-handed interventions that influence more than one variable. Can we also do this for the second causal claim made by proponents of the network theory, i.e., that environmental factors could have a direct causal impact on symptom-symptom relations? This claim is more difficult to defend, since intervening on a symptom-symptom relation would likely lead to changes in both symptoms. So, for environmental factors that are clearly temporally distinguishable from the onset of symptoms and under some interpretations of interventionism, we could potentially establish a causal relation between environmental factors and symptoms.

In response, proponents of the network theory could still explain psychiatric disorders as a system of interacting symptoms by referring to the sense-making nature of causal relations. What this section demonstrates, however, is that certain criteria should be met when trying to argue for causal relations in the network theory using interventionism. Whereas these criteria may be met for some effects of environmental factors on symptoms (given certain assumptions), it may be more difficult for others and for symptom-symptom relations. This may limit the potential of the theory to guide psychiatric practice: if it cannot provide evidence for the causal relations underlying psychiatric disorders, it limits their potential to guide psychiatric interventions. But as mentioned previously, Borsboom and colleagues also refer to constitution relations and mechanisms when describing how symptoms and environmental factors relate to psychiatric disorders. Can the network theory provide mechanistic explanations?

### Mechanistic Explanation

Mechanistic explanations are concerned with the representation of the mechanisms underlying a certain phenomenon or system, i.e., the phenomenon’s components, the components’ operations, and their causal organization (Craver and Kaplan, 2018). A mechanistic explanation of chemical neurotransmission, for example, appeals to entities (or components such as ions, neurotransmitters, vesicles, and membranes) and operations (or activities such as depolarizing, diffusing, priming, docking, and fusing) organized together so that they do something – in this case, reliably preserve a signal across the space between cells (Piccinini and Craver, 2011). Mechanistic explanation is the main explanatory strategy in the life sciences, but it does not necessarily go hand in hand with the traditional, reductionist view of psychiatric disorders. Although one could point out that mechanistic explanation is reductionist insofar as it appeals to entities and operations at a lower level of organization, mechanistic explanation does not advocate a sole focus on neurobiology. Indeed, mechanistic explanation typically involves multiple levels of organization and it does not privilege the lowest level. This means that the network theory is theoretically compatible with the mechanistic explanatory strategy, even if it does not include (neuro)biological information.^12^

Can we conceptualize environmental factors as constitutive parts of the mechanism underlying psychiatric disorders? To address this question, we can refer to discussions on the possible extension of cognitive phenomena. Some philosophers have argued that cognitive mechanisms are situated in and dependent on the environment, but that we should not consider environmental factors as part of the mechanism explaining cognitive phenomena. For example, Bechtel (2009, p. 156) states that “for mental phenomena it is appropriate to treat the mind/brain as the locus of the responsible mechanism and to emphasize the boundary between the mind/brain and the rest of the body and between the cognitive agent and its environment.” However, Craver (2007, p. 141) suggests that “many cognitive mechanisms draw upon resources outside of the brain and outside of the body to such an extent that it is not fruitful to see the skin, or surface of the central nervous system (CNS), as a useful boundary.” If we extrapolate this to psychiatric disorders, we could argue that defining them in an extended sense so that they include brain, body, and environment, allows us to explain them using extended mechanisms.

But if we argue that environmental factors and symptoms can together constitute psychiatric disorders, a different problem arises: where to draw the boundary of the disorder and the mechanism that we want to describe? Recall the example by Borsboom et al. (2019a), in which they state that gambling machines are literally part of the mechanism that explains gambling disorder. If gambling machines are part of this mechanism, why should the mechanism not also include other external entities or events, such as gambling legislation, entry tickets, or socio-cultural norms regarding gambling? Similar claims can be made for substance use disorders. Having an opioid use disorder, for example, depends heavily on the availability of opioids, but does this mean that the person who provides these drugs should be considered part of the disorder’s mechanism? These examples illustrate that claiming that environmental factors are a part of the mechanism of a psychiatric disorder raises questions on the *boundaries* of the disorder: where do we draw the line between factors that are explicitly part of the mechanism and thus constitutive for the phenomenon that we want to explain and other external factors that simply causally influence the mechanism or are preconditions for the mechanism’s emergence? Craver (2007) has proposed *mutual manipulability* as a criterion to decide whether a part or its activity is constitutively relevant for a phenomenon. According to this criterion, the behavior of a spatiotemporal part *X* of a system *S* is constitutively relevant to *S*’s behavior if, and only if, the behaviors of *X* and *S* can be mutually manipulated. Craver defines manipulability in terms of a change in behavior brought about by an intervention à la Woodward (2003). This demarcation criterion is attractive because it could potentially transform the philosophical debate about cognitive extension into a tractable, empirical debate (Kaplan, 2012). However, several philosophers have argued that Craver’s mutual manipulability condition is problematic insofar as it undermines the fundamental distinction between constitution and causation. Indeed, constitution is typically treated as a *non-causal* dependency relation between lower-level parts and higher-level mechanisms. This issue is still a subject of intense debate. To provide a definition of constitutive relationships in terms of interventionism, some have argued for the use of the fat-handed intervention criterion (Romero, 2015; Baumgartner and Gebharter, 2016; Baumgartner and Casini, 2017). Nevertheless, as demonstrated earlier, interpreting network theory in light of (fat-handed) interventionism still faces important challenges, hampering the possibility to establish mutual manipulability relations using interventionism. Hence, it is uncertain whether adding this demarcation criterion would help to decide the issue in the context of the network theory.

There is another, more pressing problem for the mechanistic explanatory potential of the network theory: in order to construe a mechanistic explanation of a phenomenon, the phenomenon should be *decomposable* in terms of components (structural decomposition) and operations (functional decomposition). Recall the example on chemical neurotransmission: this phenomenon is mechanistically explanatory because it is structurally decomposable in terms of ions, neurotransmitters, vesicles, and membranes, and functionally decomposable in terms of depolarization, diffusion, priming, docking, and fusion. Are psychiatric disorders decomposable in this sense? It has been argued that there are two types of systems with different levels of decomposability. In a *nearly decomposable* system, the behavior of the system’s individual components is integrated, but the components can still be understood and studied independently. Bechtel (2009) argues that cognitive systems are nearly decomposable, meaning that they can be explained mechanistically. In a *non-decomposable* system, the (short-term) behavior of the system’s component parts highly depends on the behavior of other individual component parts. Since no subsystems of components are (nearly) independent of one another, the system cannot be explained mechanistically (Rathkopf, 2018). It is an open-ended question which system best describes psychiatric disorders. It may be possible that psychiatric disorders are in fact nearly decomposable, and that the theory’s current description of psychiatric disorders in terms of symptoms and environmental factors provides a mechanism sketch that can be filled in with more (structural) details as more research becomes available (Piccinini and Craver, 2011). However, it may also be possible that psychiatric disorders are in fact non-decomposable. As mentioned earlier, the network theory claims that symptoms operate in causal feedback loops. If systems are characterized by circular causality, i.e., a given component of the system is both continuously affecting and simultaneously being affected by activity in another component, it is difficult to identify the contribution of the component in question in terms of the underlying structural entities (Lamb and Chemero, 2014).^13^ Even if this were possible, we still face the problem discussed previously: individual symptoms may not be as easily differentiated as commonly assumed, thereby limiting the decomposability of psychiatric disorders. If we conclude on the basis of these considerations that psychiatric disorders are in fact non-decomposable systems, we cannot explain them mechanistically and cannot substantiate the claims made by proponents of the network theory concerning constitution.

This section addressed two issues concerning the mechanistic explanatory potential of the network theory. First, we showed that there are difficulties in justifying that environmental factors *constitute* or *cause* psychiatric disorders or symptoms. Second, we can only substantiate the claim that symptoms and environmental factors co-constitute psychiatric disorders using mechanistic explanation if psychiatric disorders are in fact decomposable.^14^ This does not imply that the network theory cannot help us to explain the development and guide the treatment of psychiatric disorders. Rather, it demonstrates that it can only have mechanistic explanatory potential when certain criteria are met, and when we adopt a specific understanding of mechanistic explanation.

## The Topological Explanatory Strategy

Proponents of the network theory do not only make reference to the individual relations between factors, but also to the characteristics of symptom networks themselves. Borsboom (2017, p. 7) argues, for instance, that the psychopathology network, an interdiagnostic network including all possible psychiatric symptoms, “has a non-trivial topology, in which certain symptoms are more tightly connected than others. These symptom groupings give rise to the phenomenological manifestation of mental disorders as groups of symptoms that often arise together.” The psychopathology network thus features *clustering*, i.e., groups of strongly related nodes (Borsboom et al., 2011). However, it is also suggested that the characteristics of symptom networks can explain the development and sustenance of psychiatric disorders. Indeed, Borsboom (2017) argues that the presence of high symptom-symptom connectivity can explain the dynamics of psychiatric disorders: in symptom networks with *high connectivity*, symptoms continue to activate each other after the initial activation of one symptom. Is this claim compatible with a topological explanatory strategy?

Topological explanations explain the dynamics of complex systems by making use of topological properties, i.e., properties of a complex system that are mathematically quantified using graph theory (Kostić, 2019). To illustrate what topological properties are, a classic example might help. In their seminal publication, Watts and Strogatz (1998) used networks to examine, among others, how infectious diseases spread by studying two topological properties: the *characteristic path length* and the *clustering coefficient*. Path length refers to the number of edges (i.e., the graph-theoretical term for relations) on the shortest path between two nodes (i.e., the graph-theoretical term for variables), and the characteristic path length is defined as the average shortest path length between all pairs of nodes in the network. The clustering coefficient is a measure of the cliquishness of the network (i.e., the degree to which nodes near each other are strongly connected). Watts and Strogatz (1998) discovered empirically that many networks have high clustering coefficients and short characteristic path lengths, a topological property they called the *small-world property*. Their simulations demonstrated that the human population is like a small-world network, which explains why diseases can spread quickly throughout the population.

This example illustrates that topological properties can be used to explain the dynamics of a system constituted by interacting parts. But what exactly is meant by explain in this context?^15^ According to Kostić (2020), a topological explanation supports counterfactuals that describe a counterfactual dependency between a system’s topological properties and its network dynamics (i.e., if the topological property would not have been there, the network dynamics would have been different). He distinguishes two ways in which topological explanations may describe counterfactual dependency relations: a *vertical* explanation in which a global topological property (characteristic of the whole network) determines certain general properties of the real-world system, and a *horizontal* explanation in which a local topological property (characteristic of a part of the network) determines certain local dynamical properties of the real-world system. Kostić (2020) illustrates the difference between these two modes of explanation by focusing on the question of cognitive control, i.e., how the brain as a dynamical system efficiently transitions between internal states. If the explanation-seeking question is: “why can the brain achieve cognitive control?,” the relevant vertical counterfactual is: if the brain would not have been a small-world network, it would not have been able to achieve cognitive control. If the explanation-seeking question is: “how and why can the brain efficiently transition between states?,” one of the relevant horizontal counterfactuals is: had the local topological properties not determined the energy requirements for those transitions, then these energy requirements would have been different. How can counterfactual dependence account for explanatory asymmetry, i.e., the topological property explaining the phenomenon and not vice versa? Kostić (2020) suggests three ways in which this can be done. First, the phenomenon that the topological property wants to explain is not a mathematically quantified property, hence there is *property asymmetry*. Second, there is *counterfactual asymmetry*: the phenomenon depends on the topological property, but the topological property does not depend on the phenomenon. Third, reversing the direction of explanation makes the claim non-explanatory. If the explanation-seeking question is: “why does a system have a certain topological property,” referring to the phenomenon is not a scientifically relevant answer. Hence, there is *perspectival asymmetry*.

The claim by Borsboom (2017) concerning connectivity can be interpreted as a vertical topological explanation: a global, mathematically quantifiable property of the network (i.e., high connectivity) explains the vulnerability to develop a psychiatric disorder. If symptoms would be less strongly connected, one would be less vulnerable to developing a psychiatric disorder. Support for this counterfactual dependency has been provided by network analysis. Indeed, Borsboom (2017) refers to a within-subject study demonstrating that in MDD, altering a parameter that determines symptom network connectivity changes the network’s vulnerability: when the nodes are highly connected, this increases the likelihood that activation of one symptom leads to activation of other symptoms, making it less likely for these symptoms to disappear (Cramer et al., 2016). Relatedly, high symptom network connectivity in MDD has also been associated with having a persistent diagnosis after 2 years (van Borkulo et al., 2015). So, it is possible for the network theory to make use of topological properties that counterfactually explain the dynamics of a psychiatric disorder.

An appealing feature of topological explanations is that they can and should be used to provide explanations of non-decomposable systems (Rathkopf, 2018). To illustrate this, Rathkopf uses the topological property *edge betweenness*, i.e., the number of the shortest paths between pairs of nodes that go through that specific edge (Girvan and Newman, 2002). Betweenness is a measure of the extent to which an edge occupies a central place in the network. To compute the betweenness of an edge, the shortest path length between all pairs of nodes in the network is examined, after which it is calculated what proportion of those paths incorporate that edge. This means that betweenness applies to a single edge, but that its value indirectly refers to the rest of the graph. In this way, it combines the complex patterns of interaction into one meaningful variable with explanatory power, making the non-decomposable system “epistemically accessible” (Rathkopf, 2018, p. 72). In other words, topological explanations can provide meaningful insights into psychiatric disorders if we are not able to clearly differentiate (the activity of) their underlying components.

The topological explanatory strategy does pose some difficulties in the context of psychiatric disorders, however. First, providing the right topological explanations depends on the topological property (and the phenomenon it aims to explain) to be “approximately true” (Kostić, 2020, p. 2). We can estimate topological properties using network analysis, but as highlighted previously, we should critically examine the data and statistical methods used to substantiate theoretical claims. Second, it is not always clear what the relevant counterfactuals are for a topological explanation: would a relevant counterfactual be an instance in which the psychiatric disorder is not present at all, or if symptom severity is decreased, for example? Third, how to interpret the global and local topological properties we discover is not always straightforward. For example, a set of topological properties that is frequently examined in the context of symptom networks is measures related to *centrality*. These measures reveal the relative importance of nodes in a network structure. It has been argued, however, that they may not have meaningful interpretations in the context of psychiatry, because they come with assumptions that are not necessarily met in psychopathological networks (Bringmann et al., 2019). This especially concerns global centrality measures that depend on the network as a whole (e.g., betweenness and closeness centrality).

A final issue is that thus far, we have only focused on topological explanations of the symptom network. How could the network theory include environmental factors in its topological explanations of psychiatric disorders? One option is to assess the dynamics of the symptom network with and without the presence of a certain environmental factor (e.g., Choi et al., 2017; Hasmi et al., 2018). This option, however, only allows one to make claims on the role of an environmental factor on the symptom network as a whole, and does not suffice when we are interested in multiple environmental factors (that we do not want to average) and their interactions. Alternatively, we could include environmental factors as part of a network structure. The next section will present the *multilayer* network account of psychiatric disorders as a framework for the network theory that could accommodate topological *and* causal/mechanistic explanatory strategies.

## A Multilayer Network Account of Psychiatric Disorders

The network theory may benefit from explicitly adopting a multilayer network account of psychiatric disorders. A multilayer network can be defined as a network of networks, or a network that is comprised of multiple layers with connections between and within the layers. In recent years, statistical techniques have been developed that allow for the estimation of such networks (Kivelä et al., 2014). Multilayer networks have been used to study various complex phenomena, including social, biological, and transport systems (Mucha et al., 2010; Boccaletti et al., 2014; De Domenico et al., 2014, 2016). They are also increasingly used in network neuroscience to integrate different neuroimaging modalities (e.g., to compare the structural and functional connectivity of brain regions), or to study brain networks over different time points, among others (De Domenico, 2017; Vaiana and Muldoon, 2018). What provides these networks with an advantage over *monolayer* networks is that the latter often require data to be aggregated (for example, by means of averaging) or to be ignored. Multilayer networks can retain this information by including it in different layers, making them better suited to deal with multidimensional data and allowing for analyses that could not be performed when focusing on one layer of analysis only.

Researchers have suggested that multilayer networks should also be applied to the study of psychiatric disorders (Braun et al., 2018). However, multilayer network analysis typically requires nodes to be replicated over the different layers, which poses a problem if we want to integrate information from different scales (e.g., symptoms and environmental factors) as layers in the multilayer network structure. Fortunately, statistical techniques are available that do not require such node replication (Brooks et al., 2020). This enables the statistical estimation of multilayer networks including various different factors that are relevant to the development, sustenance and potential treatment of psychiatric disorders. It has been argued that these innovations in multilayer network analysis techniques should be paired with innovations in the *theoretical* frameworks of psychiatric disorders, doing justice to their dimensional and multiplex nature (Braun et al., 2018).

Although proponents of the network theory do not explicitly endorse a multilayer network account of psychiatric disorders, their claims are compatible with this view. More specifically, the multilayer network account provides an explicit framework for the network theory that can include multiple different factors, with the additional advantage that it can be statistically modeled.^16^ First, it is compatible with the claim that “basically every element of the system is dependent on a heterogeneous set of biological and external factors” (Borsboom et al., 2019a, p. 9). Multilayer networks provide a framework that can easily be extended to accommodate various non-symptom factors interacting with the symptom network. Second, proponents of the network theory claim that environmental factors could be part of the *mechanism* that *constitutes* symptoms or symptom-symptom relations. It may be possible for multilayer networks to account for this claim when symptoms and environmental factors are construed as different layers in the network structure.

A multilayer network account has other explanatory advantages as well, insofar as it might be able to accommodate both mechanistic/causal explanations and topological explanations of psychiatric disorders. First, a multilayer network account may enhance the mechanistic explanatory potential of the network theory, by incorporating different factors that are part of the mechanisms underlying psychiatric disorders. In this sense, the account is compatible with the claim that psychiatric disorders are *mechanistic property clusters*: clusters of properties that span multiple layers and are maintained by interacting, dysfunctional, and self-sustaining mechanisms (Kendler et al., 2011). Both accounts argue that there is not one layer that can tell us all we want to know about a psychiatric disorder: rather, complex and multi-layer causal mechanisms, including genetic, cellular, neural, psychological, environmental and socio-cultural factors produce, underlie and sustain psychiatric disorders (Kendler, 2008). However, as claimed earlier, psychiatric disorders can only be explained mechanistically if they are decomposable. Multilayer networks could include layers with a higher degree of decomposability, such as structural neurobiology (e.g., anatomical connectivity obtained with diffusion-weighted magnetic resonance imaging). Since such an underlying network could include information on concrete parts and operations (and their causal interactions), it would allow for the possibility of structural decomposition as is required by the mechanistic explanatory strategy. Structural data could also constrain functional data (e.g., Suárez et al., 2020), compatible with the mechanistic claim that function needs to be constrained by structure. One could also speculate that these layers with higher decomposability may meet more of the criteria for good interventions than purely functional layers, which means that their inclusion could allow for local causal explanations of elements of psychiatric disorders. So, a multilayer network account may enhance the mechanistic explanatory potential of the network theory, although this hinges on the issue of the decomposability of psychiatric disorders and the layers that such a theoretical framework would incorporate.

However, the multilayer network account could also enhance the explanatory potential of the network theory if psychiatric disorders turn out to be non-decomposable. More specifically, it allows for topological explanations that go beyond symptom networks. In this way, it can do justice to the idea that interactions between non-symptom factors are relevant for explaining the development, sustenance, or potential treatment of psychiatric disorders. First, topological properties of non-symptom layers may inform us about the topological properties of the symptom network. As mentioned above, high connectivity between symptoms has been related to increased vulnerability to develop psychiatric disorders. Psychiatric disorder-related changes in connectivity patterns have also been demonstrated in networks at multiple layers of brain organization (van den Heuvel and Sporns, 2019; van den Heuvel et al., 2019). So, exploring the dynamics of non-symptom layers of the multilayer network structure may provide information about the dynamics of the symptom network. Second, multilayer networks may allow for topological explanations of psychiatric disorders that span multiple layers. Indeed, statistical techniques have both extended traditional topological properties to multilayer networks and developed topological properties specific to multilayer structures (see Vaiana and Muldoon, 2018 for an overview). Such multilayer topological explanations may provide new insights into the dynamics of psychiatric disorders that supersede what we could explain if we solely focus on the symptom network. For example, De Domenico et al. (2015) demonstrated that hubs in multilayer neural networks differ dramatically from hubs in separate layers of the system, and Battiston et al. (2014) showed that two layers in a multilayer network exhibited different network properties but shared certain hubs and motifs (i.e., characteristic recurrent connection patterns). What could a multilayer topological explanation look like in the context of psychiatric disorders? A topological property that could be exploited is *community structure*, i.e., the presence of groups of nodes with strong internal and weak external connections. If time is added as a dimension to the multilayer network structure, the dynamical changes in community structure over time could be investigated. Braun et al. (2018) have suggested that this could be applied to the study of brain networks in individuals with a psychiatric disorder diagnosis to identify possible critical time points in their clinical development. In a similar fashion, examining how the (community structure of) the symptom network changes over time may explain the development of psychiatric disorders. Moreover, multilayer topological properties could be used to investigate and explain heterogeneity within psychiatric disorders by identifying subtypes with different multilayer topologies (e.g., including different symptoms and neurobiological factors; similar to a suggestion in the context of personality research Brooks et al., 2020).

This section demonstrated that adopting a multilayer network account could allow the network theory to accommodate both mechanistic/causal and topological explanations of psychiatric disorders spanning multiple layers. On such an account, the explanatory potential of the network theory does not hinge on whether psychiatric disorders are (nearly) decomposable. If psychiatric disorders or a specific layer turn out to be non-decomposable, it may still be possible to account for their dynamics using topological explanations, meaning that a multilayer network account is able to address a variety of explanation-seeking questions. Of course, more statistical and conceptual research into multilayer networks of psychiatric disorders is necessary to further explore their potential. Future research could, for example, examine which layers and relations are relevant to include in consultation with clinicians and experts by experience. Also, it should be examined how different layers can be defined, how they relate to each other, and which statistical methods would be most suited to estimate such networks using empirical datasets. Lastly, it should be assessed how a multilayer network account can translate to clinical practice, and to what extend it is compatible with existing theoretical frameworks (such as RDoC, with different domains potentially being represented as different layers of a multilayer network).

## Conclusion

This article critically examined the explanatory potential of the network theory that includes both symptoms and environmental factors. On the one hand, proponents of the network theory claim that causally interacting symptoms constitute psychiatric disorders and that environmental factors causally and mechanistically influence symptoms and psychiatric disorders in general. This suggests that the network theory could provide causal/mechanistic explanations of psychiatric disorders. However, to justify these claims, various assumptions should be satisfied. We cannot make causal claims based on network analysis alone, and determining causality using Woodward’s interventionist theory requires psychiatric disorders and their symptoms to meet criteria for suitable interventions, which may not always be possible. Moreover, providing a mechanistic account of psychiatric disorders is only possible if they are decomposable, and even then may it be difficult to formally differentiate between causal and constitutive relations. On the other hand, proponents of the network theory suggest that it might be possible to explain psychiatric disorders in terms of the characteristics of symptom networks themselves. We showed that adopting a topological explanatory strategy may be promising for the network theory, for it can explain the dynamics of psychiatric disorders when they are non-decomposable, but it does pose issues as well. Lastly, we argue that adopting a multilayer network account of psychiatric disorders provides a framework for the network theory that could accommodate different factors related to psychiatric disorders as well as both mechanistic/causal and topological explanations.

A multilayer network account differs vastly from the traditional view of psychiatric disorders we started with. Critical voices may argue that we have traded a relatively straightforward account of how to understand and explain psychiatric disorders with an overly complex alternative. Indeed, arguing that psychiatric disorders are brain disorders seems much easier than appealing to an account of psychiatric disorders that includes different types of factors and relations between layers and individual factors. However, it is unlikely that our explanations of psychiatric disorders will ultimately be simple (as demonstrated by the lack of empirical support for the traditional view). Instead of trying to reduce the complexity of psychiatric disorders, it may be preferable to embrace their complex and multifaceted nature. An account that does this while still having explanatory potential may ultimately provide a more comprehensive understanding of psychiatric disorders and more guidance for psychiatric practice. The network theory should be applauded for aiming to provide an explanatory framework that captures some of this complexity, and the multilayer network account should be seen as a possible elaboration of this theory. This does not mean that the multilayer network account is the only conceptualization of psychiatric disorder that does justice to their complexity. Nonetheless, moving toward such an account may be more fruitful for psychiatry than moving toward oversimplification.

## Data Availability Statement

The original contributions presented in the study are included in the article/supplementary material, further inquiries can be directed to the corresponding author.

## Author Contributions

NB is responsible for the final structure of the manuscript and has primarily contributed to the sections on the interpretation of the network theory, network analysis, Woodward’s interventionist theory, topological explanations, and multilayer networks. LB has primarily contributed to the sections on mechanistic and topological explanations (in multilayer networks). GG and JG have provided supervision and feedback on the argumentation. All authors contributed to the article and approved the submitted version.

### Conflict of Interest

The authors declare that the research was conducted in the absence of any commercial or financial relationships that could be construed as a potential conflict of interest.

## Footnotes

^1^In this article, we distinguish between network *theory* and network *analysis*. We use the term network analysis to refer to the statistical techniques used to estimate networks based on empirical data. This term can be used synonymously with network methodology and network psychometrics. Network theory aims to address and explain the nature of psychopathology and to give an account of what psychiatric disorders *are* (Borsboom et al., 2019b). We will discuss this distinction more thoroughly in section “The causal/ mechanistic explanatory strategy.”

^2^One of the anonymous peer reviewers alluded us to the article by Colombo and Heinz (2019) that assesses which theoretical framework can best integrate different aspects of psychiatric disorders. More specifically, they address how computational phenotypes and phenomenological information could be integrated into one explanatory account of alcohol use disorder. Similar to our article, Colombo and Heinz (2019) propose that such an integrative account should include multiple layers, and they discuss network models as one of the possibilities for explanatory integration. They argue that networks cannot include multiple layers (i.e., are *flat*), and claim that a dimensional model may be a more promising framework for explanatory integration. We agree that dimensional models may also be of interest, but it is important to note that the network theory (and the Borsboom and Cramer, 2013 article they make reference to) does not reject the possibility that a network may consist of multiple layers. This will be further addressed in section "A multilayer network account of psychiatric disorders."

^3^Borsboom et al. (2019a) claim that the relations between symptoms make sense by referring to interpretivism, i.e., the notion that we attribute beliefs, emotions, and desires with specific content to ourselves and others explain and predict behavior (Dennett, 1987). On their account, we can make sense of and understand why one symptom can lead to another by referring to their intentional content, i.e., what they are about, and people’s *basic rationality*. For example, if one beliefs they may be spreading germs, it makes sense that they wash their hands excessively, since hand washing is a reasonable strategy to prevent the spreading of germs. Issues with this interpretation have been raised (e.g., Slors et al., 2019), but discussing this goes beyond the scope of this article.

^4^This claim is not made explicitly by Borsboom (2017) or Borsboom et al. (2019a), but it has been endorsed and explained in Borsboom (2008), , Fried and Cramer (2017), and Oude Maatman (2020).

^5^The first two symptoms – depressed mood and diminished interest – are considered *core symptoms*, meaning that at least one of them needs to be present. Additionally, to receive a MDD diagnosis, the symptoms need to cause clinically significant distress and the episode should not be attributable to a substance or another medical condition or disorder.

^6^It should be noted that the notion of *disorder heterogeneity* also poses a problem for the traditional view: how can we justify referring to a common cause when there is substantial heterogeneity in the way psychiatric disorders are manifested? One possible albeit controversial means to solve this problem is to argue that different symptom manifestations constitute different disorders, but discussing this alternative in depth is beyond the scope of this article.

^7^When binary data is used, network estimation makes use of Ising models, whose edges do not correspond to partial correlations coefficients but can be similarly interpreted.

^8^Statistical tools have been developed that could account for feedback loops in causal graphs, i.e., estimate *directed cyclic graphs* (Spirtes, 1995; Richardson, 1996). However, these techniques have not (yet) been applied to symptom networks, and since their assumptions are stricter than those of DAGs, it is unlikely that these will be met in the context of psychopathology.

^9^This example was taken from de Bruin (2020).

^10^Borsboom (2017) uses the term “granularity” rather than detail and specificity, but we assume that this was implied.

^11^Interestingly, Woodward (2014) argues that multiple realizability of psychiatric symptoms (i.e., the notion that they may be realized by multiple different physical and/or neural states) could be problematic for applying interventionism to psychiatric disorders, whereas Borsboom et al. (2019a) use multiple realizability as an argument against the traditional view of psychiatric disorders (since it would hamper the possibility of reducing symptoms to brain states).

^12^Some may argue that network theory is not compatible with mechanistic explanation because of its “flatness”: mechanistic explanations require the presence of multiple layers, but the network theory does not explicate this. We will further address this notion in section “A multilayer network account of psychiatric disorders.”

^13^Note that the concept of circular causality itself has received criticism (Bakker, 2005).

^14^One could argue that (structural) decomposition is not essential for mechanistic explanation (Zednik, 2014), and that it is more important that mechanistic explanations demonstrate how phenomena are “situated in the causal structure of the world” (Craver, 2013, p. 134). However, as argued previously, demonstrating causal relationships in the context of network theory may also pose issues.

^15^Some philosophers have questioned the explanatory potential of topological properties. For example, Craver (2016) argues that topological explanations are in fact exploratory, because they cannot distinguish good from bad explanations. Moreover, as an anonymous reviewer pointed out, one could argue that topological explanations do not provide information on *why* certain topological properties, and not a relevant contrast class, yield these network dynamics.

^16^Interestingly, proponents of the network theory seem sympathetic to the idea that different factors related to psychiatric disorders may represent different network structures. Borsboom et al. (2019b) argue that psychological networks may relate to underlying biological networks, either in a part-whole relationship or with biological networks being *nested* in a symptom network. This latter statement is similar to a claim made in an earlier article, stating that “the reality of psychopathology involves a Russian doll of networks nested within networks in several layers of complexity” (Borsboom and Cramer, 2013, p. 104). Here, they argue that symptom networks could relate to networks of environmental factors (i.e., social networks) and to neurobiological networks. However, their suggestions present methodological difficulties, as it is not clear how nested networks could be modeled statistically (Borsboom et al., 2019b).
